# Socioeconomic determinants of myalgic encephalomyelitis/chronic fatigue syndrome in Norway: a registry study

**DOI:** 10.1186/s12889-024-18757-7

**Published:** 2024-05-13

**Authors:** Geir Haakon Hilland, Kjartan Sarheim Anthun

**Affiliations:** 1 SINTEF Digital, Department of Health, Health services research group, Strindvegen 4, Trondheim, 7034 Norway; 2 Department of Public Health and Nursing, Norwegian University of Science and Technology, Håkon Jarlsgate 11, Trondheim, 7030 Norway

## Abstract

**Background:**

Previous research has shown that socioeconomic status (SES) is a strong predictor of chronic disease. However, to the best of our knowledge, there has been no studies of how SES affects the risk of Myalgic encephalomyelitis/chronic fatigue syndrome (ME/CFS) that has not been based upon self-reporting or retrospectively screening of symptoms. As far as we know, this is therefore the first study that isolate and describe socioeconomic determinants of ME/CFS and calculate how these factors relate to the risk of ME/CFS diagnosis by utilizing individual level registry data. This allows for objective operationalization of the ME/CFS population, and makes it possible to model SES affect the risk of ME/CFS diagnosis, relative to control groups.

**Data and methods:**

We conduct a pooled cross-sectional analysis of registry data from all adult patients diagnosed with ME/CFS from 2016 to 2018 in Norway, coupled with socioeconomic data from statistics Norway from 2011 to 2018. We operationalize SES as household income and educational attainment fixed at the beginning of the study period. We compare the effects of SES on the risk of ME/CFS diagnosis to a population of chronically ill patients with hospital diagnoses that share clinical characteristics of ME/CFS and a healthy random sample of the Norwegian population. Our models are estimated by logistic regression analyses.

**Results:**

When comparing the risk of ME/CFS diagnosis with a population consisting of people with four specific chronic diseases, we find that high educational attainment is associated with a 19% increase (OR: 1.19) in the risk of ME/CFS and that high household income is associated with a 17% decrease (OR:0.83) in risk of ME/CFS. In our second model we compare with a healthy population sample, and found that low educational attainment is associated with 69% decrease (OR:0.31) in the risk of ME/CFS and that low household income is associated with a 53% increase (OR: 1.53).

**Conclusion:**

We find statistically significant associations between SES and the risk of ME/CFS. However, our more detailed analyses shows that our findings vary according to which population we compare the ME/CFS patients with, and that the effect of SES is larger when comparing with a healthy population sample, as opposed to controls with selected hospital diagnoses.

**Supplementary Information:**

The online version contains supplementary material available at 10.1186/s12889-024-18757-7.

## Introduction

Myalgic encephalomyelitis (ME), or chronic fatigue syndrome (CFS), is regarded as a complex and highly disabling chronic disease with currently unknown pathophysiology, which has no approved cure [1]. Those suffering from ME/CFS have a complex assortment of immunological, neurological, and psychological symptoms, resulting in high levels of comorbidity [2]. The physiological symptoms are diverse and include chronic pain, sensory hypersensitivity, and severe fatigue (e.g., post exertional malaise) [1]. These symptoms often persist in combination with psychological ailments, such as cognitive decline and depression. The burden from ME/CFS is severe both in an individual and societal perspective [3].

A systematic review of the prevalence and incidence of ME/CFS in Europe from 1994 to 2019 shows that prevalence ranges from 0.1 to 2.2% depending on diagnosis criteria, study designs and populations [4]. Some studies that calculate the incidence rate of ME/CFS by age and gender find that women are at approximately 3 times higher at risk of ME/CFS diagnosis, and that the risk is highest in the age groups 15–25 and 35–45 [5, 6]. However, it is important to note that the risk of ME/CFS diagnosis by age group and gender will depend upon various study specific factors, such as data availability and operationalization of the ME/CFS population. The comorbid and chronic nature of the disease results in a very high and sustained use of various health services from those affected by the disease, and yearly economic costs of ME/CFS has been estimated to be substantial [7]. In addition, studies find that caregivers of those affected are also highly burdened [1].

Therefore, the various aspects of ME/CFS should be understood as much as possible. Foremost to reduce the suffering of those afflicted by ME/CFS and their caregivers, but also to lower the societal costs associated with the disease. The disease is not well enough understood to implement effective preventative measures and treatments, especially when it comes to those that are moderately or severely afflicted by it. As of today, there is a lack of studies about the relationship between SES and ME/CFS, even though we know that socioeconomic status is a strong predictor for the onset of an array of diseases, especially diseases with comorbid facets and complex pathologies between somatic and psychological symptoms.

We argue that the relationship between SES and ME/CFS is understudied and that the few studies that have been conducted on the subject have utilized data that are prone producing biased results, mainly stemming from a small number of participant and self-reporting. In addition, there is a rather unique problem of selection bias when studying ME/CFS due to the lack of medical tests and biomarkers for the disease. This is an essential factor that underpins the whole discourse, one that is often overlooked in individual studies of the disease. The lack of objective medical tests results in heterogeneity challenges in defining the ME/CFS populations in epidemiological studies, which in turn reduce the transferability of findings from studies on ME/CFS populations across study settings (e.g., across time or between countries). This further strengthens the rationale for our study of the relationship between SES and the risk of ME/CFS in Norway. We contribute to the ME/CFS discourse trough this paper, where we study the relationship between socioeconomic status (SES) and the risk of receiving ME/CFS diagnosis in the Norwegian population, using comprehensive registry data.

## Previous research

### The relationship between socioeconomic status and chronic disease

Socioeconomic status can be defined in several ways. We follow Psaki and colleagues’ [8] definition of the concept:

*“Socioeconomic status (SES) is a theoretical construct encompassing individual, household, and/or community access to resources. It is commonly conceptualized as a combination of economic, social, and work status, measured by income or wealth, education, and occupation, respectively”* [8].

Several studies find correlations between low SES and various health outcomes, including a higher risk of chronic diseases, the disease group of which ME/CFS is a part of [9, 10]. It is therefore vital to account for the multifaceted theoretical construction of SES when operationalizing the concept for quantitative analyses. Not doing so, for instance if operationalizing SES as *only* income, or *only* educational attainment, could lead to results with questionable validity. Diemer at al [11]. problematize the utilization of incomplete SES measures and reviews the literature for best practice when operationalizing SES. They conclude that the “gold standard” of measuring SES as an objective quantification of social class involves three parts: occupation, educational attainment, and income [11].

This conceptualization of SES does not capture subjective social status (SSS), which is also a relevant predictor for both physical and mental health [12, 13]. SSS is often explored using qualitative data to gain knowledge of the subjective aspect of social standing. It is however outside the scope and objectives of this study, and we therefore explicitly focus on objective social class affiliation operationalized as various aspects of quantifiable SES.

### The relationship between socioeconomic status and ME/CFS

To our knowledge, the first study of the link between SES and the risk of ME/CFS was initiated by the U.S Centre for Disease Control (CDC) in the latter part of the 1980s and published in 1993 [14]. In this epidemiological study, the authors concluded that highly educated Caucasian women, with the potential for high income, comprised most of the ME/CFS cases. However, later studies did not find statistically significant differences between various socioeconomic strata and the risk of developing ME/CFS [15–17]. The findings in the discourse are divergent, as other studies again *do* find that SES status is a predictor for ME/CFS. Interestingly, several studies find that high SES status is a predictor [18–20], which contradicts research on the relationship between SES and the onset of chronic disease in general [21]. Other studies find that the onset of ME/CFS is associated with middle-to-low socioeconomic status, which contradicts these findings [22, 23].

In sum, the literature shows that socioeconomics potentially influences the probability of receiving an ME/CFS diagnosis, even though these variables alone far from adequately explains the onset of the disease. Nevertheless, research shows that further understanding of the socioeconomic status of people with ME/CFS is needed to understand the relationship between socioeconomics and the disease especially given the challenges posed by selection bias when studying ME/CFS.

## Data and methods

### Study design and population

This study was designed as a pooled cross-sectional analysis where we analysed the probability for ME/CFS diagnosis in the Norwegian population by pooling (1) a random sample of the Norwegian population and (2) a population consisting of individuals diagnosed with chronic diseases that share clinical characteristics with CFS/ME, and (3) the ME/CFS population. The identification criteria of ME/CFS prior to washout was patients with the diagnosis registered in the Norwegian patient register at least once in the years 2016–2018.

One of the major challenges when studying how different factors relate to the risk of ME/CFS diagnosis is the disease’ long and complex diagnostic process. Therefore, our study was designed with an early measurement/exposure of SES, combined with a long ME/CFS “washout” period of 5–7 years. All individuals with ME/CFS diagnosis prior to 2016 was excluded from our sample. This long washout period considers the complex diagnostic process of ME/CFS and reduce the probability that factors related to the onset of the disease affects the levels of SES for each individual. As the objective of our study was to test how levels of SES prior to ME/CFS diagnosis affects the probability of diagnosis, we modelled how the 2011 values of our SES variables affected the probability of receiving an ME/CFS diagnosis 5–7 years later. This way, the effects of ME/CFS onset on each individual’ values of SES (i.e., reverse causality) was reduced.

**Table 1:** Inclusion and exclusion into populations

Inclusion/ Exclusion	Random sample	Chronic control	ME/CFS
Inclusion	Randomly selected by Statistics Norway as 1/1000 of population	Patients with hospital episodes in 2009–2018 at least once with at least one of the diagnoses A69.2, C50, G35 or M97.9	Patients registered with a G93.3 diagnosis at least once in hospitals in 2016–2018
Exclusion	Patient being part of chronic or ME/CFS group	Patient being part of ME/CFS group	Patients with at least one registered diagnosis of G93.3 in the years 2009–2015

The random sample of the healthy population was created by randomly selecting 0.1% of the total Norwegian population from the registries. As we wanted to study the effect of SES on the risk of ME/CFS relative to a healthy, randomly selected population, we removed all individuals with hospital diagnoses in the Norwegian patient registry (NPR). The chronically ill patient population was created by drawing individuals registered in NPR with the following ICD-10 diagnoses: C50 “Malignant neoplasm of breast”, M79.7 “Fibromyalgia”, G35 “Multiple sclerosis”, and A69.2 “Lyme disease”. These diagnoses were chosen because they include other chronically diseases with increased disease burden for a long but limited time frame. In addition, they either share clinical characteristics with ME/CFS (e.g., fatigue and idiopathic pain) and/or disproportionately affect women, which previous research has shown is the case also for ME/CFS [5, 6]. Any overlap of either random sample or hospital controls and ME/CFS-patients were classified as ME/CFS, and any overlap of random sample and hospital control were classified as hospital controls.

### Data sources

We tested the relationship between SES and the risk of ME/CFS diagnosis by utilizing individual level registry data from two sources. Hospital visits and diagnoses were collected from the Norwegian Patient Register (NPR), and SES-related data were collected from Statistics Norway. 5 556 individuals with ME/CFS were included in the study after washout, 60 425 individuals with one of the included control diagnoses were included, and finally 5 562 individuals were randomly drawn from the population.

### Study variables

We follow Diemer and colleagues [11] and operationalize SES as comprised of both income level and educational attainment. This approach is also supported in other studies, for instance by Callahan & Eyberg [24] which found that a model containing separate variables for income and educational attainment explained three times more of the variance observed in the outcome of study [11]. Therefore, instead of utilizing composite measures such as indexes that collapse different SES categories, we model the effect of each SES variable separately, which is argued to be the best approach to preserve the individual effects of each SES variable [25, 26]. Unfortunately, we do not have the data that would be required test occupation as a SES-indicator.

### Dependant variable: ME/CFS

We tested how the 2011 values of our independent variables predicted the risk of receiving an ME/CFS diagnosis 5–7 years later through logistic regression analyses. We modelled how socioeconomic status affected the probability of receiving a ME/CFS diagnosis (ICD10, G93.3) in specialized healthcare. Our outcome variable was dichotomous with the value of 0 if a patient did not receive G93.3 and 1 if an individual in our dataset had a ME/CFS diagnosis.

### Household income

We operationalized income as a categorical variable consisting of three categories, consisting of low income, medium income and high income. Low income was defined as household income below 40% of the median household income, whilst high income was defined as above 70% of the median. We use the medium household income as a reference category, as we are interested in analysing the associations between high and low income and the risk of ME/CFS diagnosis.

### Educational attainment

Educational attainment was constructed at the family level as the highest level of education for either the individual, or either of the parents in 2011. We measured educational attainment as a categorical variable consisting of three categories: low education, medium education, and high education. Low education was defined as having no education or having completed only pre-school and elementary school. Medium educational attainment was defined as having completed lower secondary school, secondary school or lower university degree, whilst high educational attainment was defined as having completed a university degree, at masters or Phd level. Medium educational attainment was the reference category in our regression analyses.

### Control variables

In addition to our independent SES variables, we controlled for occupational status, marital status, gender and age. Occupational status is operationalized as a dichotomous variable where the effect of being employed in 2011 is included in the model. Marital status is operationalized as a categorical variable measured in 2011, and we model the effect of being married and separated/divorced relative to being single. Gender is operationalized as a dichotomous variable and the effect of female sex is included in the model. Age is a categorical variable consisting of 7 categories measured in 2018, and the effect of each age group is modelled relative to the 0–17 age group. Our variables are operationalized as categories, and Table 2 shows the distributions of individuals in each category of our variables. Table 2 presents unweighted data, while the regressions have age and gender weights to standardize.

**Table 2:** Distribution (percentage) per category of each variable, by gender and population

	Category	Hospital controls		Random sample		ME/CFS	
		Men	Women	Men	Women	Men	Women
Household income	Low	5.3	6.5	4.8	4.8	7.2	10.3
	Medium	86.1	86.4	88.2	91.6	86.4	85.6
	High	8.7	7.1	7.0	3.6	6.5	4.1
Educational attainment	Low	8.7	5.4	32.7	31.7	13.8	12.3
	Medium	82.7	89.2	61.3	62.8	75.1	80.4
	High	8.6	5.5	6.0	5.5	11.1	7.3
Working	Not working	45.2	43.6	56.4	57.7	52.2	46.8
	Working	54.8	56.4	43.6	42.3	47.8	53.2
Marital status	Single	38.4	24.1	55.0	48.3	75.1	65.6
	Married	48.3	52.6	35.8	36.0	19.6	23.9
	Divorced /separated	13.3	23.3	9.3	15.7	5.3	10.5
Age	Age 0–17	5.0	0.7	20.9	20.0	20.6	8.9
	Age 18–24	2.1	1.2	8.5	8.7	14.5	17.0
	Age 25–34	8.6	5.8	14.5	12.7	18.6	20.5
	Age 35–44	16.1	11.9	13.6	13.4	18.6	23.9
	Age 45–54	23.8	22.3	14.1	14.2	17.3	20.1
	Age 55–64	20.4	23.1	11.5	12.4	8.6	7.9
	Age 65+	24.1	35.1	16.9	18.7	1.8	1.8
	N	7 806	52 619	2 884	2.678	1 206	4 350

### Statistical analyses

We estimated the association between SES and ME/CFS by conducting a logistic regression analysis. The equation of our baseline logistic model, where they key explanatory variables are household income and educational attainment, can be written as.

$${L_i}\, = \,{\beta _0}\, + \,{\beta _1}\,{X_{1i}}\, + \,{\beta _2}\,{X_{2i}}\, + \,{\beta _k}\,{X_{k,\,i}}$$

The total logit (L_i_) was a linear function of the *X*-variables, and ***k*** the number of parameters included in our model (the constant and all *X* variables). As previous research has shown that there are strong effects of gender on the risk of ME/CFS, we included interaction terms between gender and both household income and educational attainment. Z_i_ denotes the interaction term, where the effect of coefficients β_x_ Z_i_ for Y, regressed on X at values of the moderator Z_i_:

$${L_i}\, = \,{\beta _0}\, + \,{\beta _1}\,{X_{1i}}\, + \,{\beta _2}\,{X_{2i}}\, + \,{\beta _x}\,{Z_i}\, + \,{\beta _k}{X_{k,\,i}}$$

As all our covariates were categorical, we estimated the effect of each category relative to a given reference category for each variable. We conducted a Hosmer–Lemeshow goodness-of-fit test [27]. The results from the goodness of fit test were statistically insignificant, which is an indication of reasonable model fit. To reduce the threat of heteroskedasticity we estimated all models using Huber-White robust standard errors [28, 29]. All statistical analyses were conducted in Stata, version 16 [30].

Since the different populations have different age and sex distributions, we have created weights for direct standardization of age and sex. The weights used in model 1 and in model 2 differ since there are different populations included in the models.

## Results

In Table 3 we present our results in a joint table consisting of two models. The table also presents the crude odds ratios.

**Table 3:** Logistic regression analyses of the relationship between SES and ME/CFS. Estimations are relative to hospital diagnosed controls (Model 1) and a healthy randomly selected sample of the Norwegian population (Model 2). Crude odds ratio, full model adjusted odds ratio and 95% confidence interval

	Model 1	Model 1	Model 1	Model 2	Model 2	Model 2
	Crude OR	Full model odds ratio	95% confidence interval	Crude OR	Full model odds ratio	95% confidence interval
Low education	1.643	0.903	0.770–1.060	0.287	0.307	0.266–0.354
High education	1.440	1.188	0.996–1.418	0.941	1.004	0.831–1.213
Low household income	1.455	1.046	0.899–1.217	1.928	1.534	1.269–1.853
High household income	0.608	0.825	0.678–1.004	0.868	1.181	0.945–1.477
Working	0.999	0.970	0.874–1.078	1.251	0.751	0.662–0.854
Married	0.304	0.766	0.683–0.858	0.611	0.843	0.732–0.970
Divorced/separated	0.339	1.045	0.901–1.211	0.818	1.206	0.983–1.481
Women	0.482	0.847	0.765–0.938	4.300	4.180	3.787–4.614
Age 18–24	1.282	1.022	0.799–1.308	3.170	1.194	1.001–1.424
Age 25–34	0.318	0.269	0.210–0.345	2.287	0.891	0.732–1.085
Age 35–44	0.166	0.150	0.116–0.193	2.695	0.906	0.737–1.114
Age 45–54	0.088	0.080	0.062–0.103	2.246	0.679	0.545–0.847
Age 55–64	0.033	0.031	0.023–0.041	1.078	0.314	0.246–0.402
Age 65+	0.006	0.005	0.004–0.007	0.163	0.042	0.031–0.056
Constant		1.335	1.025–1.738		1.139	0.975–1.332
Observations		64,548			10,065	
Pseudo R^2^		0,1477			0,1465	

Model 1 estimates ME/CFS relative to hospital controls, model 2 estimates ME/CFS relative to random healthy population. Crude odds ratios were calculated from unadjusted models. Reference categories were medium education, medium income, not-working, not-married, men, age 0–17. Exponentiated coefficients

In model 1 we compared the risk of ME/CFS diagnosis with a population consisting of people with four specific chronic diseases. Our findings in model 1 showed that there was a statistically significant effect from low educational attainment and the risk of ME/CFS. Our models showed that high educational attainment increased the risk of ME/CFS by 19% (OR: 1.19), relative to medium educational attainment. We found a negative but not statistically significant effect from low educational attainment. There was a statistically significant relationship between household income and risk of ME/CFS diagnosis in Model 1. We found that high household income decreases the risk of ME/CFS by 17% (OR: 0.825). There was no effect from low household income in model 1. In model 1, we found no association of employment (OR 0.97). Being married was associated with a 23% decrease (OR: 0.766) in risk of ME/CFS diagnosis, relative to being not married. Furthermore, when comparing with the population consisting of chronically ill individuals, we found that women have an 15% reduced risk of ME/CFS diagnosis (OR: 0.85). This reduced risk is likely due to the selection of breast cancer as a control group, so that even though women have a higher risk over all (see Table 2) for ME/CFS, when comparing with the four diagnostic groups women are at lower risk. This must not be interpreted as a reduced risk for women, but a relatively lower risk than for other chronic diseases that is more prevalent amongst women than men. For age, we modelled the risk of ME/CFS per age group relative to the 0–17 group. When comparing with the hospital diagnosed population sample, a population that skewed strongly towards older age, we saw that the risk of ME/CFS was highest in the age group 18–24 years, as the risk of ME/CFS was strongly reduced in all other age groups in the model.

In model two, we modelled the risk of ME/CFS relative to a healthy, randomly selected sample of the Norwegian population. We found a statistically significant relationship between educational attainment and risk of ME/CFS diagnosis, relative to the healthy population sample. Low educational attainment was statistically significantly associated with a 68% reduction (OR: 0.307) in the risk of ME/CFS relative to medium educational attainment, while there was no statistically significant relationship between high educational attainment and the risk of ME/CFS. The result from our logistic regression in model 2 showed a statistically significant relationship between low household income and the risk of ME/CFS (OR: 1.53) relative to medium household income. Low household income increased the risk of ME/CFS by 53%, relative to belonging to the medium household income group. Employment is associated with decreased risk of ME/CFS, as individuals that were employed had a 25% (OR = 0.75) reduction in risk of ME/CFS diagnosis. We found that being married decreased the risk of ME/CFS diagnosis by 16% (OR: 0.84). Furthermore, we found that women had a 4.2 time (OR: 4.18) greater risk of G93.3 diagnosis than men. For age, we modelled the risk of ME/CFS per age group relative to the 0–17 group. When comparing with the healthy random population sample, we saw that the risk of ME/CFS was highest in the age groups 18–24 (OR: 1.19).

**Fig. 1:**
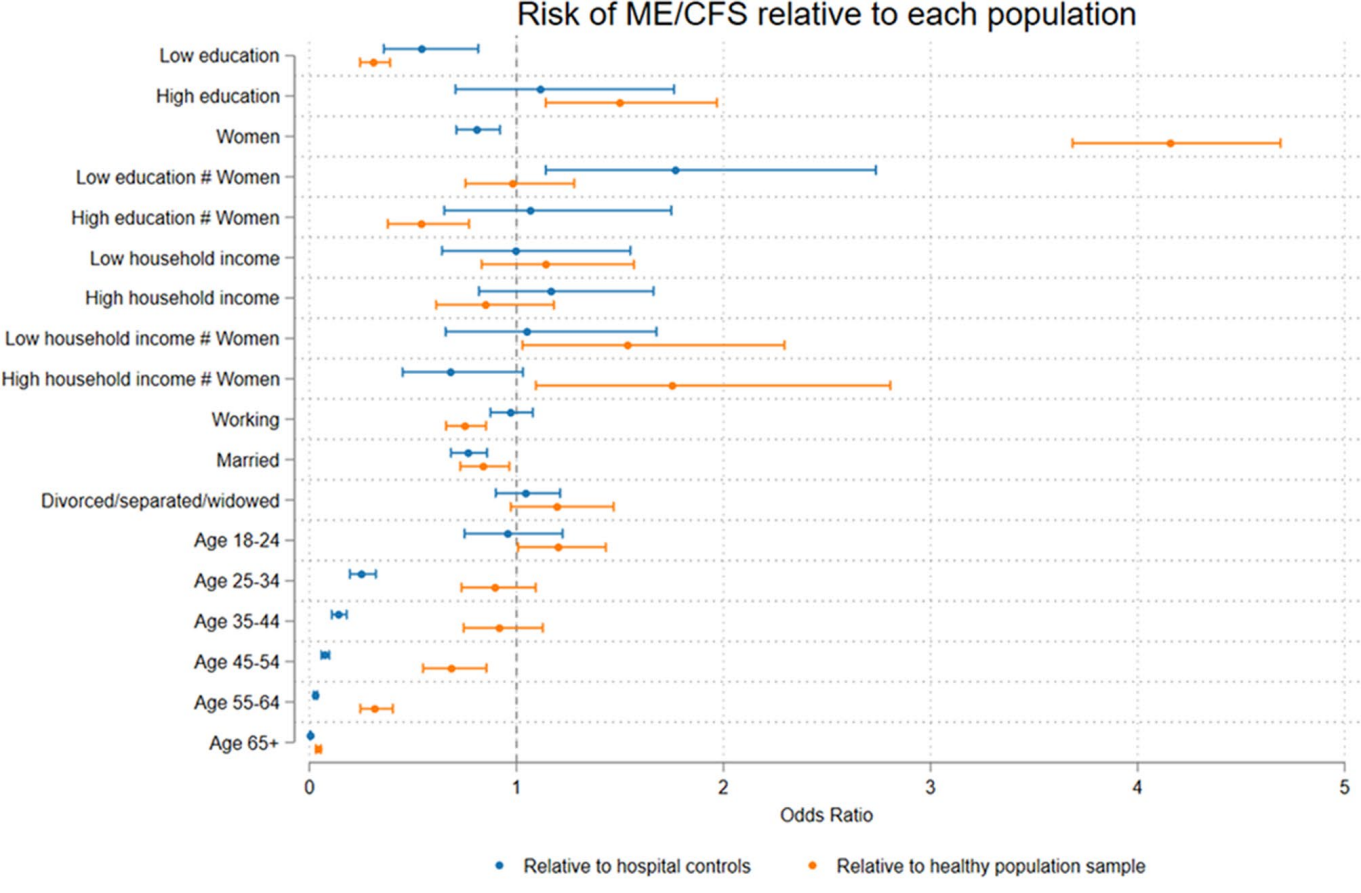
Confidence intervals for model 1 and model 2. Risk of ME/CFS relative to a healthy population sample and relative to hospital diagnosed controls

We also tested interactions of sex, education and income. Regression results are available in the supplementary materials. Figure 1 shows the confidence intervals from each variable in the analysis from models including interaction effects.

### Robustness tests

We conducted four robustness tests to account for the strong effects of gender and age on the risk for ME/CFS diagnosis. Firstly, we estimated the effect of age as a cubic spline, secondly we estimated interactions of sex and the main independent variables, and thirdly we also estimated models stratified on sex. Finally we removed the washout period for diagnostic inclusion to test if the results depended strongly on the washout.

Cubic splines of age were included to test if the effect of age would be better fitted as a non-linear continuous association. It has been argued that cubic spline interpolation is imperative in some cases to achieve a good fit between a continuous variable such as age, and a dichotomous outcome such as ME/CFS diagnosis [31]. Our tests indicated that while there were strong drawbacks from modelling age either as a continuous variable, there were no improvements in fit from modelling age as a cubic spline as opposed to a categorical variable consisting of 7 categories. We therefore present our findings with age as a categorical variable as these results are much more intuitive to interpret for the reader, as opposed to cubic spline knots.

We also tested if there were interaction effects between gender and our two SES variables (see supplementary materials tables A1, figures A1 and A2), as previous research has shown that there are strong effects from gender on the risk of ME/CFS diagnosis (cf [5]). The model showed that both education and income interacted with gender. However, the interactions were barely statistically significant and only for specific age groups, which makes it hard to give meaningful interpretations of the interaction effects. We therefore only included the interaction effect in the appendix.

Thirdly, we also performed stratified analysis by gender. These are attached in appendix in tables A2 and A3. These results allow us to separately look at the associations for men and women. Overall, results are very similar to the main model presented in Table 3. We see that the association for income is mainly associated through women and not men, while the difference for educational attainment is more driven by men.

Finally, we removed the washout period for diagnostic inclusion. Results are presented in appendix in table A4. This analysis included a total of 9 323 unique patients with ME/CFS diagnosis, of which 5 556 (59.6%) got the diagnosis first in the years 2016–2018, in other words slightly higher number than what was already included in the models. We split the analysis in four groups^1^ depending on the onset of ME/CFS. We have primarily focused on model 1, i.e. a comparison to other chronic conditions. Our results show that the first recorded diagnosis in 2016–2018 does not significantly deviate from the other categories due to large confidence interval, but that the effect of socio-economic variables depend on the washout period: aside from low household income, all other three indicators became stronger with the longer washout period.

## Discussion

The findings presented in model one suggests that high household income decreases the risk of ME/CFS by 17% (OR: 0.825) relative to medium household income, when comparing to a control group consisting of individuals with hospital diagnosis that share clinical characteristics of ME/CFS. There was no effect from low household income. We also found a negative but not statistically significant effect from low educational attainment in model one. However, we do find a statistically significant effect from high educational attainment in model one, which increased the risk of ME/CFS by 19% (OR: 1.19). In model two, where we compare the ME/CFS population with a randomly selected sample of the Norwegian population, we found that low educational attainment was statistically significantly associated with a 68% reduction (OR: 0.307) in the risk of ME/CFS relative to medium educational attainment. We found no statistically significant relationship between high educational attainment and the risk of ME/CFS. Furthermore, the result from our logistic regression in model two showed a statistically significant relationship between low household income and the risk of ME/CFS (OR: 1.53) relative to medium household income.

There are two important factors that underpin the entire discussion of our findings, which is important to present before we discuss the results of our analyses. The first factor is related to how we operationalize ME/CFS in this study. We only include ME/CFS patients that are diagnosed in the specialized healthcare services in Norway with the ICD-10 code G93.3 which excludes all individuals that are either only diagnosed in primary care or that suffer from undiagnosed ME/CFS.

Secondly there is a lack of biomarkers for ME/CFS, rendering traditional medical tests unusable and resulting in ME/CFS having to be diagnosed clinically by the individual physicians, based on a combination of various diagnostic tools or guidelines and subjective assessments of the patient’ symptoms. As we discussed in the introduction, this opens for a problem of selection bias due to heterogeneity stemming from differences in both individual patients and consulting physicians. This also increases the possibility of misdiagnosing individuals with ME/CFS. Taken together, these factors leave the interpretation of our findings vulnerable to type I and II errors, if one disregards these constraints posed by the operationalization of ME/CFS as a hospital diagnosis and the problem of selection bias that stems from clinically diagnosing the disease. However, we do believe that our study design reduces the probability of misinterpreting our findings, as we will discuss in this chapter.

We operationalized SES as educational attainment and household income. For educational attainment, the findings of our analyses diverged from the established discourse on the relationship between education level and the risk of chronic disease in general. Taken at face value, there seems to be a reverse effect of education for ME/CFS as we find that low educational attainment reduces the risk of ME/CFS diagnosis, relative to medium educational attainment, when comparing with the healthy population sample controls. However, our analyses do not indicate that this is an epidemiological effect of low educational attainment that is unique for the risk of ME/CFS, rather it seems to reflect the relationship between educational attainment and the risk of chronic disease in general. When comparing with a population consisting of hospitalized controls this effect is already accounted for in the model, and we see that the effect of low education is reduced to 10% and becomes statistically insignificant (95% CI [0.77–1.06]). However, the effect of low educational attainment is close to being statistically significant and we argue that this suggests that there are effects of educational attainment and the risk of ME/CFS, even when comparing with a population that innately controls for the effect of educational attainment on the likelihood of hospital diagnosis. This finding should be taken together with the fact that we do find a statistically significant increase in risk of ME/CFS diagnosis for people with high educational attainment when comparing with hospital diagnosed controls.

This suggests that there is an effect of educational attainment on the risk of ME/CFS diagnosis, even when comparing with a population that innately controls for the existing relationship between SES and chronic disease in general. It is, however, unlikely that this is an epidemiological effect of educational attainment on the risk of ME/CFS, though it is theoretically possible given how poorly the disease is understood. What is more likely, is that given the unique characteristics of ME/CFS, specifically the lack of medical tests and the need for clinical diagnosis that naturally follows from the lack of objective tests and biomarkers, the cohort consisting of people with low educational attainment is probably less likely to receive an ME/CFS diagnosis, compared to people with medium educational attainment. Individuals from families in the low educational attainment group or individuals with low educational attainment does not necessarily have a reduced risk of the disease as a function of their education level. Our findings could rather be interpreted as that they are less able to ask for, or less likely to receive, a G93.3 diagnosis in meeting with the specialized healthcare system.

Previous research has shown that clinical perceptions of patients with low SES affects the decisions made by clinicians in meeting with their patients [32]. Given the historical stigma surrounding ME/CFS, this bias might be uniquely present for this disease. Furthermore, the patients with low educational attainment are likely to have lower degrees of health literacy. Previous research has shown that low levels of health literacy is correlated with less knowledge about medical conditions [33] and asking fewer questions during medical visits [34]. Previous research has also shown that people with higher SES are more likely to ask for and receive diagnoses compared to people with low SES [32].

A large study gives further credence to this interpretation [22]. Jason and colleagues screened a random sample of 18 675 individuals from 1995 to 1998 for ME/CFS symptomatology, and their analyses showed that almost 90% of people with middle to lower SES that were eligible for ME/CFS when retrospectively screening their symptoms, did not receive ME/CFS diagnosis by a physician. Therefore, it is highly possible that our findings reflect the underlying differences between high and low levels of education in meeting with the healthcare services, and not an epidemiological effect of education in and of itself. Furthermore, our analyses could reflect unequal access to healthcare services, as previous research has shown that higher SES is associated with better healthcare access [32, 35]. The finding of an association between low educational attainment and reduced likelihood of ME/CFS diagnosis could therefore reflect unequal access to healthcare services. However, it is less likely that this effect is very prominent in our analyses, given the fact that Norway has a publicly funded healthcare system focused on egalitarianism. Nevertheless, it is a factor that could theoretically explain this finding.

This explanation becomes less likely as we do not find similar pattern in terms of the effect of household income. In fact, our findings related to household income reflect the large body of research on the relationship between wealth and the risk of chronic disease in general. For income, our analyses indicated that the same mechanisms that explain the relationship between income and reduced risk of chronic illness in general is prevalent for the risk of ME/CFS as well. When we compare to a randomly sampled population, we find that low household income increases the risk of ME/CFS by 53%. When comparing with a population consisting of hospitalized controls, we find no statistically significant effect of comparatively low household income, but high household income reduces the risk of ME/CFS, relative to medium household income when comparing with the hospital diagnosed control population. These findings are in line with previous research on the relationship between SES and the risk of disease in general, and specifically research on the effect of wealth on the risk of chronic illness [36].

It is important to note that we do not interpret our findings as evidence of substantial underdiagnosis of ME/CFS for people with low educational attainment in Norway as this explorative and rather descriptive study is not designed to draw this conclusion. Nevertheless, we argue that the rather unique clinical characteristics of ME/CFS, the lack of objective medical testing and the stigma surrounding the disease increases the likelihood that low educational attainment affects the clinical perceptions of these patients, which could reduce the probability of diagnosing these patients with ME/CFS. Taken together with the known effects of health literacy and healthcare access on the likelihood of hospital diagnosis, this could explain our findings. However, more research is needed to establish whether this is the case. As previously stated, our findings could also reflect an unknown epidemiological effect of low educational attainment that protects individuals from ME/CFS, or that the effect of age is not adequately captured in our models despite passing robustness checks for how we fit the effect of age in our models. We therefore encourage more research on the topic of healthcare utilization for the ME/CFS population as the implications for the healthcare system are important. If it is the case that individuals with low educational attainment are less likely to receive ME/CFS diagnosis due to factors related to health literacy and clinical perceptions of low SES patients on the part of physicians then this needs to be addressed in order to avoid underdiagnosing individuals with ME/CFS.

### Strengths and limitations

To the best of our knowledge, this is the first study about the relationship between SES and the risk of ME/CFS that utilize individual level health registry data. Individual level registry data allows for analyses with high internal validity due to objective measures for ME/CFS diagnosis, as opposed to small-N studies that rely on self-reporting, thus being prone to selection bias. The Norwegian registry data also gives us the opportunity to assess the relationship between SES and risk of ME/CFS diagnosis relative to both healthy and hospital diagnosed controls, which increase the validity of our findings. An additional strength related to our data is the fact that we can utilize a long “washout” period and model the effect of SES several years before ME/CFS diagnosis is confirmed. This way, the effects of ME/CFS on SES, i.e. reverse causality, is almost completely removed. We measure SES at the family/household level, which also reduces problems of measuring income and education for young cohorts. Another strength of our study is that we operationalize SES as separate variables instead of a composite measure. Research shows that the mechanisms behind the correlation between high socioeconomic status and good health are diverse and complex. Operationalizing SES status should be done meticulously to capture the different mechanisms, which can be problematic.

As with all studies, ours also has weaknesses. One weakness is the lack of detailed occupational data that corresponds with the required washout period for our SES variables. It would be very interesting to include this as a SES variable as previous research has shown that occupational status, operationalized as profession or types of professions, is an important socioeconomic risk factor for chronic disease. However, the most important weakness to highlight in this study, is the fact that we can only operationalize and define the ME/CFS population as the ICD-10 code G93.3. This means that all patients that are diagnosed by a primary care physician is not included in our analyses. Also, for this reason we do not include any individuals with *only* self-reported ME/CFS in this study, but only patients with diagnosis confirmed by specialist care (i.e. hospital physicians) were eligible for inclusion. This creates a form of selection bias that may lead us to underestimate the number of ME/CFS patients, as have been discussed both in this paper in previous research. We know that there is a risk of excluding immigrants and people with low societal status when studying the relationship between SES and chronic illness, as they are less likely to receive hospital diagnosis due to worse healthcare access and less resources in general [37]. This effect may even be more prevalent for ME/CFS, specifically due to the lack of biomarkers for the disease. Also, our identification of ME/CFS-patients was only for those with the diagnosis in 2016–2018, followed by an extraction of data from 2009 to 2018, so we had no information on other ME/CFS-patients in the years prior to 2016 if they did not receive the diagnosis in 2016–2018. This could cause some minor selection effect in the estimates, but since the onset of ME/CFS is likely to reduce income or delay education this study has opted to have a long washout. Previous studies have demonstrated that a longer washout yield better estimates of incidents and reduce overestimates [38, 39].

## Conclusion

To the best of our knowledge, this is the first study that utilizes individual level registry data to test the relationship between socioeconomic status and the risk of being diagnosed with ICD-10 diagnosis G93.3 (Myalgic encephalomyelitis/Postviral fatigue syndrome). The key takeaway from our analyses is that the effect of SES on the risk of ME/CFS diagnosis varies according to which population we use as a basis of comparison. We find a statistically significant relationship between SES operationalized as educational attainment and household income and the risk of ME/CFS diagnosis in all models. When comparing with a population consisting of the four selected hospital diagnosis, we find that high household income reduces the risk of ME/CFS diagnosis, while high educational attainment increases the risk. When comparing with a random sample of the Norwegian population, low educational attainment becomes statistically significant and strongly predicts a decreased risk of ME/CFS diagnosis. Furthermore, when comparing to a random population sample, we find that low household income increases the risk of ME/CFS diagnosis.

While our study confirms that SES affects the risk of ME/CFS, there is a need for more research on ME/CFS in general, and specifically on the mechanisms behind the relationship between SES and ME/CFS. Future research on SES and ME/CFS should seek to further unveil these mechanisms, for instance by including subjective social status (SSS) and more detailed occupational data as SES variables. More research on the healthcare utilization of ME/CFS patients is also needed, to further understand how SES variables such as educational attainment affects the diagnostic process pre- ME/CFS diagnosis.

## Electronic supplementary material

Below is the link to the electronic supplementary material.

Supplementary Material 1

## Data Availability

Data used in this study are available from national registries pending approval and application. We are authorized to publish findings from these data as per Norway’ Helseforskningsloven § 35. Linked health data from registries is classified as highly sensitive, and the authors of this study are therefore not authorized to share our data with the public due to the sensitive nature of individual health records.

## Footnotes

1 Similar results were found for model 2 (not shown), and also in a multinominal logistic regression (not shown).
