# Correction: Socioeconomic determinants of myalgic encephalomyelitis/chronic fatigue syndrome in Norway: a registry study

**DOI:** 10.1186/s12889-024-19908-6

**Published:** 2024-09-04

**Authors:** Geir Haakon Hilland, Kjartan Sarheim Anthun

**Affiliations:** 1 SINTEF Digital, Department of Health, Health services research group, Strindvegen 4, Trondheim, 7034 Norway; 2 Department of Public Health and Nursing, Norwegian University of Science and Technology, Håkon Jarlsgate 11, Trondheim, 7030 Norway

**BMC Public Health (2024) 24:1296**

10.1186/s12889-024-18757-7

The original publication of this article contained an error in the "Data and Methods" section. The incorrect and correct information is listed in this correction article. The original article has been updated.

Incorrect

Medium educational attainment was defined as having completed lower secondary school and secondary school, whilst high educational attainment was defined as having completed a university degree, at any level.

Correct

Medium educational attainment was defined as having completed lower secondary school, secondary school or lower university degree, whilst high educational attainment was defined as having completed a university degree, at masters or Phd level.

