# Supplementary material for: Socioeconomic determinants of myalgic encephalomyelitis/chronic fatigue syndrome in Norway: a registry study
Source: BMC Public Health. 2024 May 13;24:1296. doi: 10.1186/s12889-024-18757-7 (PMC11089728; doi:10.1186/s12889-024-18757-7)
Supplement: Supplementary file 1 — Supplementary Material 1 [file 12889_2024_18757_MOESM1_ESM.docx]

Supplementary materials

**
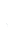

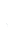

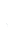

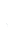

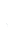

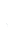

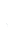

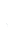

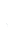

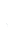
Table A1: Logistic regression analyses of the relationship between SES and ME/CFS, including interaction terms. Estimations are relative to hospital diagnosed controls (Model 1) and a healthy randomly selected sample of the Norwegian population (Model 2). Odds ratio and 95 % confidence intervals.**

|  | **1: Risk of ME/CFS relative to a hospital diagnosed controls** | | **2: Risk of ME/CFS relative to a healthy population sample** | |
| --- | --- | --- | --- | --- |
|  | **OR** | **95 % CI** | **OR** | **95 % CI** |
| Low educational attainment | 0.542 | 0.360 - 0.816 | 0.309 | 0.245 - 0.390 |
| High educational attainment | 1.115 | 0.706 - 1.762 | 1.499 | 1.141 - 1.968 |
| Women | 0.808 | 0.710 - 0.921 | 4.158 | 3.686 - 4.690 |
| Women with low educational attainment | 1.767 | 1.142 - 2.736 | 0.982 | 0.754 - 1.279 |
| Women with high educational attainment | 1.067 | 0.651 - 1.748 | 0.540 | 0.379 - 0.771 |
| Low household income | 0.997 | 0.641 - 1.551 | 1.142 | 0.832 - 1.567 |
| High household income | 1.167 | 0.819 - 1.662 | 0.850 | 0.612 - 1.181 |
| Women in low income households | 1.050 | 0.658 - 1.676 | 1.536 | 1.029 - 2.295 |
| Women in high income households | 0.681 | 0.450 - 1.032 | 1.752 | 1.094 - 2.806 |
| Working | 0.971 | 0.874 - 1.079 | 0.750 | 0.660 - 0.853 |
| Married | 0.766 | 0.684 - 0.858 | 0.839 | 0.729 - 0.966 |
| Divorced / separated | 1.044 | 0.901 - 1.211 | 1.196 | 0.973 - 1.470 |
| Age 18-24 | 0.957 | 0.749 - 1.223 | 1.201 | 1.008 - 1.432 |
| Age 25-34 | 0.251 | 0.196 - 0.321 | 0.896 | 0.735 - 1.093 |
| Age 35-44 | 0.140 | 0.109 - 0.180 | 0.917 | 0.745 - 1.127 |
| Age 45-54 | 0.0743 | 0.0575 - 0.0961 | 0.685 | 0.549 - 0.855 |
| Age 55-64 | 0.0286 | 0.0217 - 0.0378 | 0.315 | 0.246 - 0.403 |
| Age 65+ | 0.00475 | 0.00338 - 0.00666 | 0.0418 | 0.0311 - 0.0564 |
| Constant | 1.492 | 1.133 - 1.966 | 1.141 | 0.965 - 1.349 |
| N | 64 548 |  | 10 065 |  |
| Pseudo R^2^ | 0,1480 |  | 0,1480 |  |

**
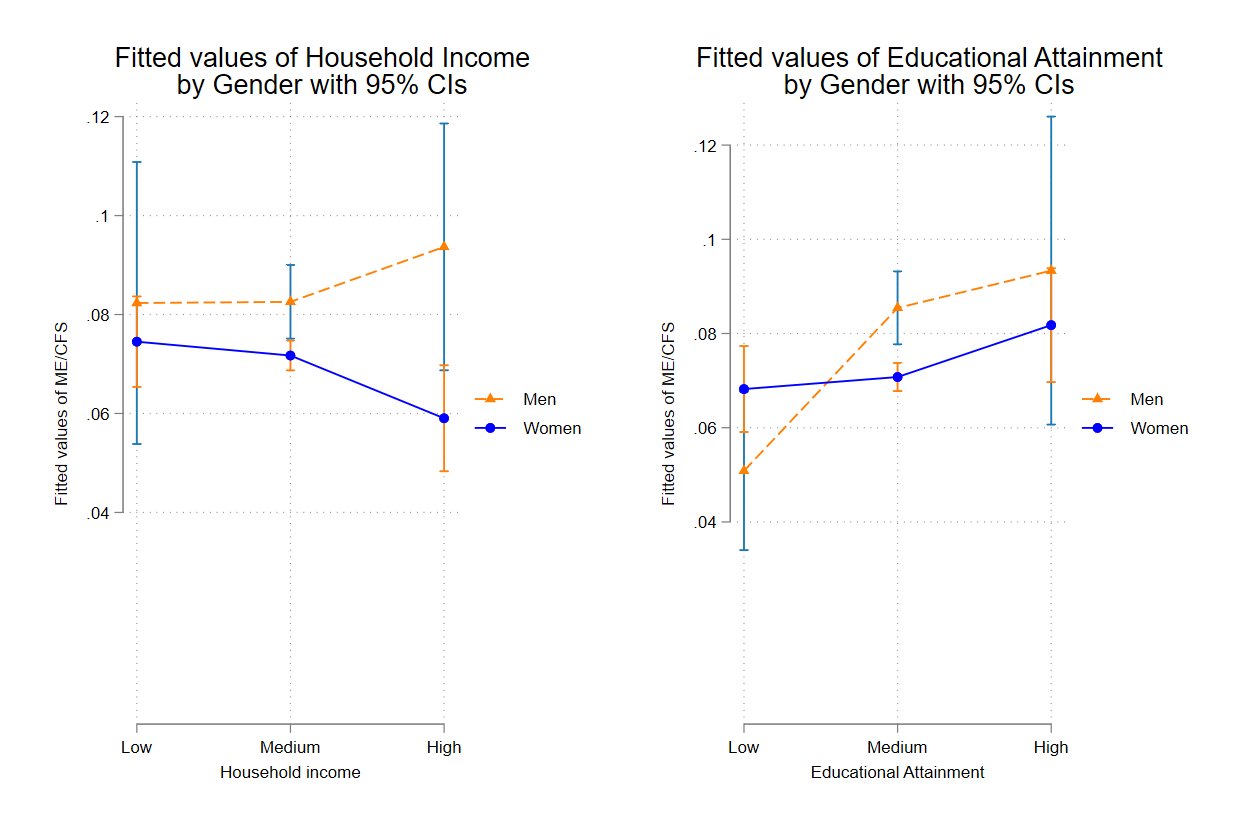
**

**Figure A1:** **Predicted marginal effects of household income and educational attainment on risk of ME/CFS diagnosis by gender, for the hospital diagnosed control population.**


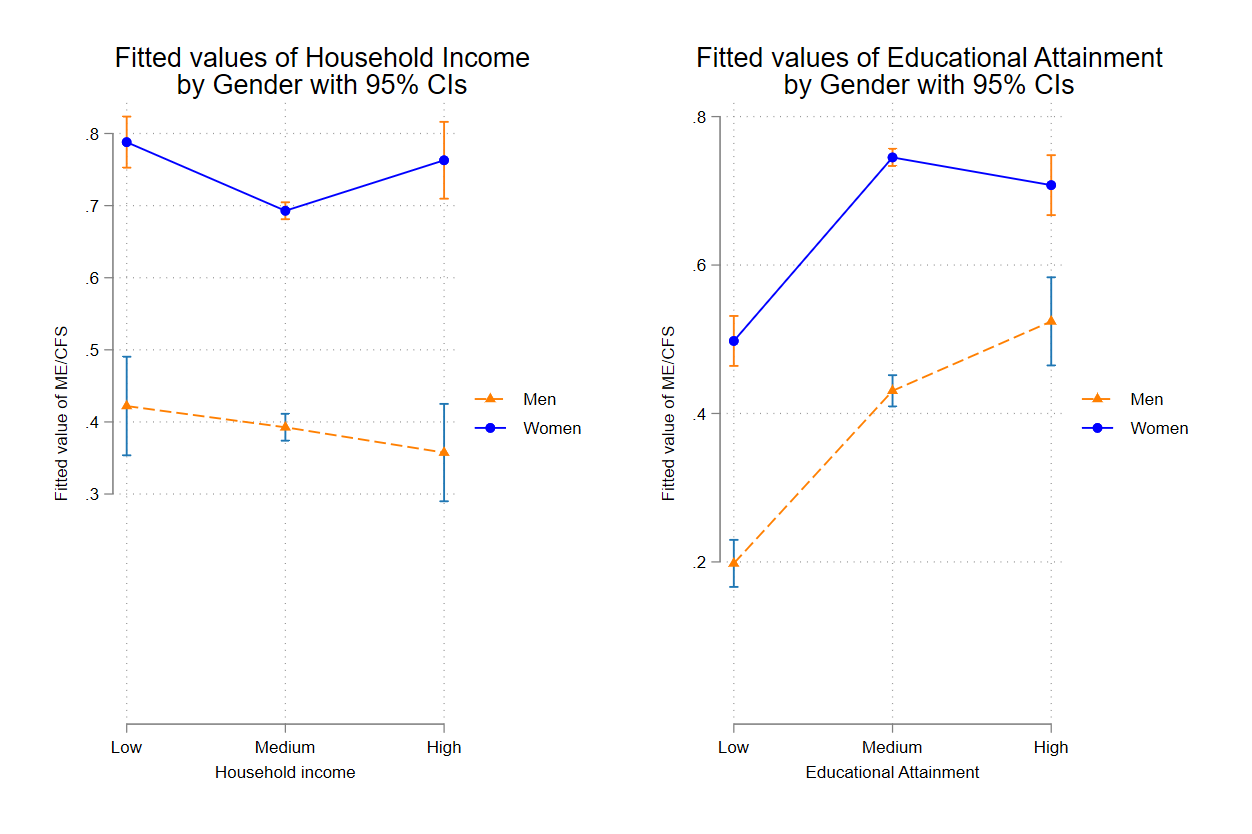


**Figure A2: Predicted marginal effects of household income and educational attainment on risk of ME/CFS diagnosis by gender, for the healthy randomly sampled control population.**

**
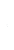

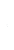

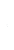

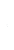

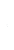

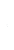

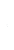

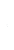

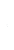

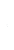
Table A2: Logistic regression analyses of the relationship between SES and ME/CFS, for men. Estimations are relative to hospital diagnosed controls (Model 1) and a healthy randomly selected sample of the Norwegian population (Model 2). Odds ratio and 95 % confidence intervals.**

| **Stratified analysis for men** | **1: Risk of ME/CFS relative to a  hospital diagnosed controls** | | **2: Risk of ME/CFS relative to a  healthy population sample** | |
| --- | --- | --- | --- | --- |
|  | **OR** | **95 % CI** | **OR** | **95 % CI** |
| Low educational attainment | 0.718 | 0.481 - 1.070 | 0.198 | 0.145 - 0.272 |
| High educational attainment | 1.279 | 0.856 - 1.911 | 1.362 | 1.036 - 1.790 |
| Low household income | 1.105 | 0.721 - 1.693 | 1.183 | 0.853 - 1.640 |
| High household income | 1.038 | 0.724 - 1.487 | 0.835 | 0.601 - 1.161 |
| Working | 1.333 | 1.096 - 1.622 | 0.809 | 0.643 - 1.019 |
| Married | 0.779 | 0.628 - 0.966 | 0.760 | 0.591 - 0.979 |
| Divorced/separated | 0.810 | 0.582 - 1.127 | 0.951 | 0.640 - 1.412 |
| Age 18-24 | 1.223 | 0.796 - 1.878 | 0.643 | 0.488 - 0.847 |
| Age 25-34 | 0.309 | 0.200 - 0.477 | 0.323 | 0.226 - 0.461 |
| Age 35-44 | 0.172 | 0.110 - 0.267 | 0.364 | 0.253 - 0.524 |
| Age 45-54 | 0.107 | 0.0681 - 0.167 | 0.311 | 0.212 - 0.455 |
| Age 55-64 | 0.0734 | 0.0453 - 0.119 | 0.191 | 0.126 - 0.290 |
| Age 65+ | 0.0110 | 0.00572 - 0.0211 | 0.0228 | 0.0131 - 0.0397 |
| Constant | 0.936 | 0.632 - 1.387 | 2.484 | 1.922 - 3.211 |
| N | 8,679 |  | 3,537 |  |
| Pseudo R^2^ | 0,1480 |  | 0,0904 |  |

**
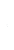

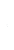

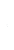

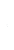

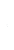

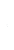

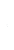

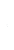

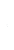

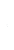
Table A3: Logistic regression analyses of the relationship between SES and ME/CFS, for women. Estimations are relative to hospital diagnosed controls (Model 1) and a healthy randomly selected sample of the Norwegian population (Model 2). Odds ratio and 95 % confidence intervals.**

| **Stratified analysis for women** | **1: Risk of ME/CFS relative to a  hospital diagnosed controls** | | **2: Risk of ME/CFS relative to a  healthy population sample** | |
| --- | --- | --- | --- | --- |
|  | **OR** | **95 % CI** | **OR** | **95 % CI** |
| Low educational attainment | 0.933 | 0.787 - 1.107 | 0.345 | 0.292 - 0.407 |
| High educational attainment | 1.186 | 0.981 - 1.434 | 0.826 | 0.655 - 1.042 |
| Low household income | 1.041 | 0.888 - 1.219 | 1.688 | 1.318 - 2.162 |
| High household income | 0.800 | 0.643 - 0.995 | 1.494 | 1.062 - 2.103 |
| Working | 0.956 | 0.856 - 1.067 | 0.731 | 0.624 - 0.857 |
| Married | 0.765 | 0.679 - 0.862 | 0.848 | 0.714 - 1.007 |
| Divorced/separated | 1.058 | 0.908 - 1.234 | 1.251 | 0.979 - 1.598 |
| Age 18-24 | 0.538 | 0.435 - 0.665 | 1.821 | 1.472 - 2.253 |
| Age 25-34 | 0.140 | 0.113 - 0.174 | 1.598 | 1.258 - 2.031 |
| Age 35-44 | 0.0779 | 0.0620 - 0.0978 | 1.557 | 1.213 - 1.998 |
| Age 45-54 | 0.0411 | 0.0327 - 0.0516 | 1.110 | 0.851 - 1.448 |
| Age 55-64 | 0.0155 | 0.0120 - 0.0199 | 0.460 | 0.343 - 0.616 |
| Age 65+ | 0.00251 | 0.00180 - 0.00350 | 0.0642 | 0.0453 - 0.0910 |
| Constant | 2.195 | 1.814 - 2.655 | 2.989 | 2.523 - 3.540 |
| N | 55,869 |  | 6,528 |  |
| Pseudo R^2^ | 0,1420 |  | 0,0822 |  |

**Table A4: Logistic analysis of the relationship between SES and ME/CFS, estimations are relative to hospital diagnosed controls (model 1). Odds ratio, and low and high band of confidence intervals.**

| **Socio- economic  variable** | **Estimate** | **Risk for no ME/CFS or onset <= 2011** | **First recorded diagnosis 2012-2013** | **First recorded diagnosis 2014-2015** | **First recorded diagnosis 2016-2018** | **Model 1 results** |
| --- | --- | --- | --- | --- | --- | --- |
| Low education | OR | 1.077 | 1.061 | 0.963 | 0.907 | 0.903 |
| Low education | Lo | 0.938 | 0.789 | 0.729 | 0.779 | 0.770 |
| Low education | Hi | 1.236 | 1.427 | 1.273 | 1.057 | 1.060 |
| High education | OR | 0.869 | 1.097 | 1.128 | 1.141 | 1.188 |
| High education | Lo | 0.750 | 0.803 | 0.835 | 0.959 | 0.996 |
| High education | Hi | 1.007 | 1.498 | 1.525 | 1.356 | 1.418 |
| Low household income | OR | 0.998 | 1.007 | 0.829 | 1.061 | 1.046 |
| Low household income | Lo | 0.878 | 0.718 | 0.647 | 0.915 | 0.899 |
| Low household income | Hi | 1.136 | 1.413 | 1.062 | 1.231 | 1.217 |
| High household income | OR | 1.163 | 0.928 | 0.976 | 0.827 | 0.825 |
| High household income | Lo | 0.986 | 0.640 | 0.686 | 0.682 | 0.678 |
| High household income | Hi | 1.370 | 1.345 | 1.389 | 1.004 | 1.004 |
| Observations |  | 67 869 | 67 869 | 67 869 | 67 869 | 64 548 |
| Pseudo R^2^ |  | 0.146 | 0.067 | 0.085 | 0.131 | 0.148 |
